# VIBES: a workflow for annotating and visualizing viral sequences integrated into bacterial genomes

**DOI:** 10.1093/nargab/lqae030

**Published:** 2024-04-04

**Authors:** Conner J Copeland, Jack W Roddy, Amelia K Schmidt, Patrick R Secor, Travis J Wheeler

**Affiliations:** Division of Biological Sciences, University of Montana, Missoula, MT, 59812, USA; R. Ken Coit College of Pharmacy, University of Arizona, Tucson, AZ, 85721, USA; Division of Biological Sciences, University of Montana, Missoula, MT, 59812, USA; Division of Biological Sciences, University of Montana, Missoula, MT, 59812, USA; R. Ken Coit College of Pharmacy, University of Arizona, Tucson, AZ, 85721, USA

## Abstract

Bacteriophages are viruses that infect bacteria. Many bacteriophages integrate their genomes into the bacterial chromosome and become prophages. Prophages may substantially burden or benefit host bacteria fitness, acting in some cases as parasites and in others as mutualists. Some prophages have been demonstrated to increase host virulence. The increasing ease of bacterial genome sequencing provides an opportunity to deeply explore prophage prevalence and insertion sites. Here we present VIBES (Viral Integrations in Bacterial genomES), a workflow intended to automate prophage annotation in complete bacterial genome sequences. VIBES provides additional context to prophage annotations by annotating bacterial genes and viral proteins in user-provided bacterial and viral genomes. The VIBES pipeline is implemented as a Nextflow-driven workflow, providing a simple, unified interface for execution on local, cluster and cloud computing environments. For each step of the pipeline, a container including all necessary software dependencies is provided. VIBES produces results in simple tab-separated format and generates intuitive and interactive visualizations for data exploration. Despite VIBES’s primary emphasis on prophage annotation, its generic alignment-based design allows it to be deployed as a general-purpose sequence similarity search manager. We demonstrate the utility of the VIBES prophage annotation workflow by searching for 178 Pf phage genomes across 1072 *Pseudomonas* spp. genomes.

## Introduction

Bacteriophages (phages), viruses that infect bacteria, are as ubiquitous as their hosts. They are found everywhere that we find populations of bacteria, from forest soils and the oceans to hydrothermal springs and the human gut. Phages pose a significant threat to bacteria: in marine ecosystems, up to one-third of bacteria are killed by phages every day (1). The strong pressure exerted by the threat of phage infection has led bacteria to evolve antiphage defense systems. Restriction–modification systems (2) and CRISPR–Cas systems (3) rely on sensing and degrading invading nucleic acids and are among the most widespread antiphage systems; however, numerous additional antiphage defense systems utilizing diverse mechanisms have recently been discovered (4). Likewise, phages have evolved to become adept manipulators of host metabolism, allowing them to evade host defenses or improve conditions for viral replication (5).

Phages can be purely parasitic (lytic) and replicate at the expense of their bacterial hosts. However, in addition to lytic replication, temperate phages can alternatively undergo lysogenic replication in which the phage genome typically integrates into the host chromosome as a prophage. Temperate phages are common: approximately half of sequenced bacterial genomes contain at least one prophage (6). Prophages are replicated each time the host cell divides. Consequentially, prophages benefit from thriving hosts, which can incentivize the development of mutualistic phage–host relationships. Many prophages encode factors that benefit their hosts (7). For example, some phages carry genes that promote resistance to infection from competing viruses (8), while other phages encode virulence factors, aiding host pathogenicity (9).

Many bacterial species are lysogenized by filamentous phages in the Inoviridae family (10). The opportunistic pathogen *Pseudomonas aeruginosa* is frequently lysogenized by an inovirus called Pf (11–13). Pf prophages maintain lysogeny by repressing transcription of their excisionase (14). In response to oxidative stress (15), nutrient limitation (16) or other factors (17), the Pf prophage excises from the chromosome and Pf virion replication is initiated. Pf virion replication plays a role in biofilm development by lysing cells in the center of a colony, releasing DNA that adds to biofilm structural integrity (18). Pf virions themselves also act as structural components in *P. aeruginosa* biofilms, protecting bacteria from desiccation and antibiotics (19,20). Indeed, the presence of Pf virions in the airways of cystic fibrosis patients is associated with antibiotic resistance (21). Additionally, Pf virions are immunomodulatory and induce maladaptive antiviral immune responses that promote infection initiation (22) and interfere with wound healing by inhibiting keratinocyte migration (23).

The important and varied roles of prophages in bacterial communities—parasites, mutualists and sometimes pathogenicity aides—motivate the development of high-quality software methods that identify and classify their integrations into bacterial genomes. Generally, phage sequence annotation tools are designed to annotate either prophage in whole bacterial genome sequence or phage genome fragments in metagenomic datasets. Most recent approaches focus on annotation in a metagenomic context, as metagenomic datasets have proven to be rich sources of previously unknown viral sequences (10,24). Viral annotation, particularly in metagenomic contexts, requires tools to strike a balance between sensitivity and speed. High sensitivity is necessary to overcome high mutation rates in viral proteins combined with an increased risk of sequencing error stemming from the low abundance of viral sequences in most metagenomic datasets. Meanwhile, reasonable labeling speed is required when annotating large datasets. As a result of these constraints, viral annotation software has generally converged on a few techniques for identifying viral sequences: sequence similarity search against large databases of viral genomes (25–27), machine learning approaches based on statistical features such as *k*-mer frequencies, with a recent emphasis on neural networks (28–30), or some combination of both approaches (31–35). Viral annotation tools with a focus on prophage annotation in whole genome sequence [DBSCAN-SWA (26), PHASTEST (36), Prophage Hunter (33)] typically include visual summaries that increase the legibility of their output. Both DBSCAN-SWA and Prophage Hunter are available as stand-alone software that can be run by researchers seeking to conduct large-scale analysis in a cluster or cloud computing environment, but require installation of prerequisite software and manual creation of job submission scripts to run in high-performance computing (HPC) environments. This significantly increases the minimum computational skillset necessary to conduct large-scale analysis of prophage integrated into complete bacterial genome sequences. PHASTEST somewhat alleviates this by offering a containerized version of their approach, reducing the need to install prerequisites. However, managing widescale execution of containerized PHASTEST instances in an HPC environment through job submission scripts or other customized approaches is still left to end users.

Here, we introduce and describe VIBES (Viral Integrations in Bacterial genomES), an automated command line workflow for annotation of bacterial genomes that emphasizes identification of prophage integrations. VIBES supplements standard bacterial gene labeling with in-depth analysis of prophage integrations, producing machine- and human-readable text output files coupled with interactive HTML visualizations that facilitate further analysis of output data. VIBES is designed to

- annotate prophages with high sensitivity;
- annotate bacterial genes on input genomes, using Prokka (37);
- annotate viral genes within input viral genomes, using the PHROGs database (38);
- accept a potentially large number of bacterial genomes and candidate phage genomes as input;
- substantially reduce prerequisite installation and automatically manage distribution of workload to cluster/cloud/local resources; and
- create interactive HTML visuals that display the above, as well as display which regions that map to user input prophages are most prevalent among all input bacterial genomes.

To the best of our knowledge, VIBES is the first prophage annotation software approach that includes output visualizations designed to facilitate investigation of patterns of prophage integration and shared prophage–host homology across entire target bacterial genome datasets. VIBES is also the first prophage annotation approach that is engineered to enable efficient, massively parallelized prophage search and genome annotation by end users on a wide variety of hardware and computing environments. VIBES also has the capacity to serve as manager of massively parallelized sequence similarity searches, even if the queries and targets of those searches are not prophages and bacteria, respectively.

## Materials and methods

### Implementation

**Figure 1.**
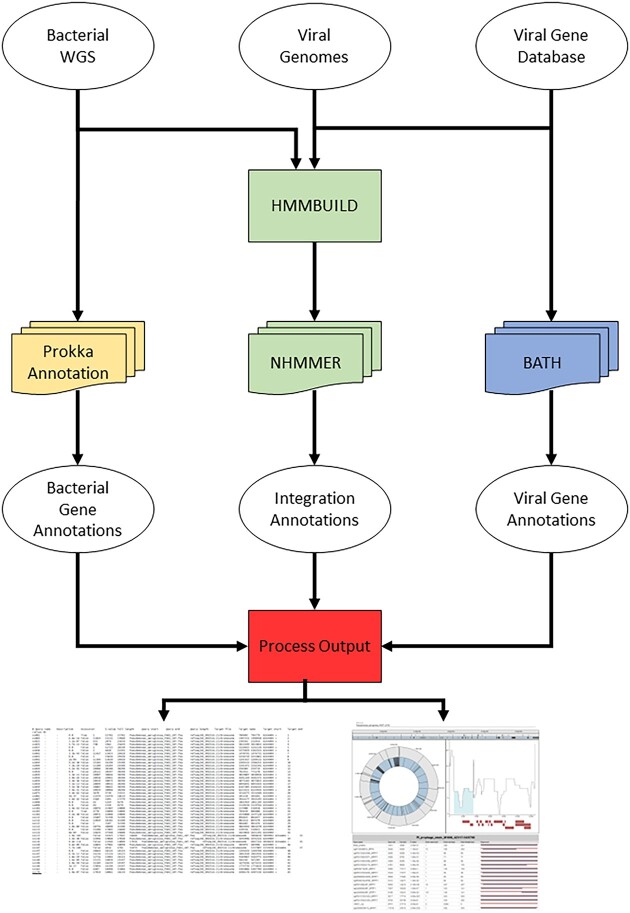
VIBES workflow schematic. It displays how input data move through the VIBES annotation workflow. Bacterial gene annotation processes are shown in yellow (left track), prophage annotation processes are shown in green (center track), viral gene annotation processes are shown in blue (right track) and visualization processes are shown in red (bottom square). The annotation processes are independent of each other. Stacked icons indicate processes parallelized automatically by Nextflow.

VIBES is an automated prophage search workflow that uses containerized components coordinated by the Nextflow workflow management software (39) to produce output tab-separated value (TSV) annotation tables accompanied by interactive HTML files that summarize matches to prophage sequences. To annotate prophage integrations, VIBES is provided with an input FASTA file containing all prophage sequences to seek and a collection of bacterial genomes in FASTA format to annotate with prophages; it performs search using the software *nhmmer* (40). To annotate bacterial protein-coding genes, ribosomal RNA and transfer RNA, VIBES uses Prokka (37). To annotate query prophage protein-coding genes, VIBES uses the BATH protein-coding DNA annotation software (41) and the PHROGs v4 prokaryotic viral protein database (38) by default. The user can optionally substitute their own viral protein database. Figure 1 provides a visual representation of the three independent annotation workflows, run in parallel and managed by VIBES, that produce bacterial gene annotations, viral sequence integrations and viral gene annotations. Table 1 lists the tools and databases managed by the VIBES Docker container.

**Table 1.** Dependencies managed by VIBES Docker container

Tool	Purpose
BATH (41)	Annotation of protein-coding DNA on query prophage
*nhmmer* (40)	DNA-to-DNA identification of prophages on bacterial genomes
Prokka (37)	Bacterial genome annotation

Before running VIBES, the user must install a software container system such as Docker (42) or Singularity/Apptainer (43) (usually the latter on HPC systems, where VIBES is likely to be utilized). These container systems enable the development and release of portable and reproducible software environments with fine-grained control over configuration and dependency conflicts while also retaining high performance. The user must also install the workflow management software Nextflow. Nextflow manages downloading and running containers, submits jobs to compute cluster job scheduling software (i.e. SLURM) or cloud computing architectures (i.e. AWS Batch), caches and checkpoints in-progress jobs in case of a crash, provides interpretable workflow status updates, runs on a wide variety of operating systems and hardware configurations, and can run VIBES locally as needed. After a user configures the workflow to run on their system and launches it, Nextflow requires no further user interaction to identify task dependencies, automatically maximizing parallelism by running as many tasks with satisfied dependencies as available resources allow.

The VIBES release consists of a Nextflow workflow script, several helper scripts written in Python and Perl, JavaScript and HTML files that produce the visualizations, and a Docker image that manages the internal configuration and dependency map of multiple tools. VIBES software and workflow can be found at https://github.com/TravisWheelerLab/VIBES.

### VIBES components

#### Prophage search component

The primary component of the VIBES workflow is its prophage search. This component searches for user-provided query prophage sequences within bacterial genomes to identify prophage integrations. Identification of prophage within bacterial genomes is performed using a DNA sequence annotation tool, *nhmmer* (40), with default settings. User queries are supplied to *nhmmer* via either a multi-FASTA file containing single sequences or a Stockholm-formatted file containing multiple sequence alignments. When provided with multiple sequence alignment queries, VIBES automatically generates query profiles. Though *nhmmer* is slower than *blastn* (44), *nhmmer*’s improved sensitivity in the face of high sequence divergence and neutral mutation (40) is useful in the context of prophages, which can mutate at rates comparable to single-stranded RNA viruses during lytic replication (45) and may show substantial divergence from query sequences. In general, any matches to a query prophage that fail to meet an *E*-value threshold (1e−5 by default) are discarded. Multiple matches to a single target sequence are adjudicated using a method borrowed from the *dfamscan* adjudication script used in Dfam (46): when multiple hits are detected with overlap >75%, the highest-scoring match is retained.

**Figure 2.**
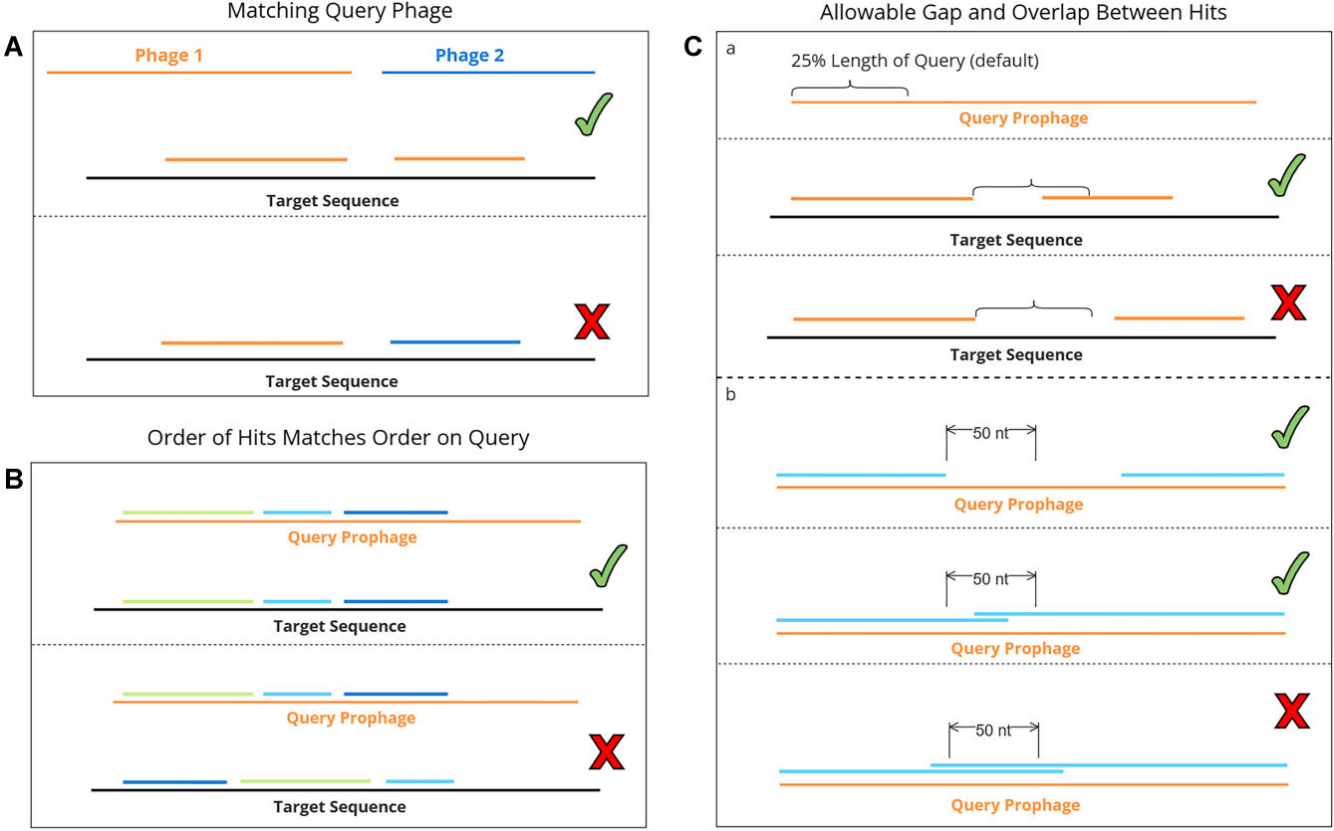
All conditions that must be met for consecutive matches to be joined (assigned the same integration ID and displayed as one integration in visual output). (**A**) Joined candidates must be assigned to the same query phage. (**B**) Joined candidates must occur in the same order on both the query phage and target bacterial genomes. (**Ca**) Given a query phage genome of length *n*, a match that ends at position *i* on the bacterial genome and a consecutive match that begins at position *j*, two matches are considered near enough for fragment joining if |*i* − *j*| ≤ *n* × *k*, where *k* is a fragment gap threshold value set to 0.25 by default. (**Cb**) Given a fragment whose match to the query viral genome ends at position *s* and a consecutive fragment whose match to the query viral genome begins at position *t*, the fragments are joined only when |*t* − *s*| ≤ *θ*, where *θ* is a constant set to 50 by default. Large gaps between matches on the query prophages are not penalized, as they may represent large deletions.

Sequence annotation tools such as *nhmmer* frequently produce fragmented alignments when presented with sequences highly diverged from the query sequences, particularly when a match contains a large inserted or deleted element relative to its nearest query. As a result, single prophage integrations may be reported as several fragments that lie close to each other on a bacterial genome in *nhmmer* output. To address these potentially fragmented integrations, VIBES includes a post-processing step that examines every match detected on a single bacterial genome, looking for consecutive matches that satisfy all of the following potential fragments must match to the same query phage sequence (Figure 2A), occur in the same order on the bacterial genome as on the query (Figure 2B), be close to each other on the bacterial genome (Figure 2Ca) and overlap minimally on the query phage (Figure 2Cb). Gaps between two matches on the query prophage sequence are not penalized, as they may represent large deletions.

#### Gene annotation of both viral and bacterial genomes

VIBES provides supplementary context to identification and investigation of prophage integration sites by identifying protein-coding genes in both full bacterial genomes and query prophage sequences. Each bacterial genome is annotated using the annotation tool Prokka (37) via StaPH-B’s Docker image (47), supporting gene annotation without requiring users to download or set up sequence databases. Like the prophage search component, each bacterial genome is annotated independently of other genomes, allowing Nextflow to fan out as many parallel Prokka annotation tasks as resources permit.

VIBES also produces gene annotations for the user-provided prophage sequences with its viral protein-coding gene annotation component. This component uses a translated search tool, BATH (41), to search a viral protein database against prophage DNA sequences. Translated search tools like BATH do not penalize neutral mutations that change DNA sequences without modifying the encoded protein sequence, making them especially well suited to annotating sequences with high mutation rates, such as viral genomes. BATH’s translated search is also robust to frameshift-inducing insertions or deletions, which can confound other translated search tools. By default, VIBES uses the PHROGs v4 viral gene database (38) reformatted as a BATH-compatible HMM database, but users can substitute other amino acid sequence or HMM databases as desired.

Although VIBES was developed with annotating prophage integrations in mind, it is primarily a framework for managing and parallelizing runs of *nhmmer*, Prokka and BATH with some prophage-annotation-specific features (the default PHROGs database is phage-specific and the VIBES-SODA visualization suite assumes query sequences are prophage). In particular, the prophage search component simply searches for matches to a query database (prophages by design) in a set of target genomes (bacteria by design) and can easily be repurposed by providing the workflow with a non-phage query sequence file and a set of non-prokaryotic genomes. Likewise, the Prokka bacterial gene annotation and BATH translated amino annotation components can be used to orchestrate massively parallel protein-coding sequence annotation, even on datasets where prophage integrations are not of interest.

#### Interactive visual generation

To facilitate further analysis and improve human readability of results, VIBES produces dynamic annotation visualizations in HTML files that can be opened in a web browser. These visuals depict prophage annotations, bacterial gene annotations and viral gene annotations. After all other workflow tasks are complete, VIBES generates a collection of HTML files, each of which contains a dynamic visualization built with the SODA sequence annotation visualization library (48). The visualizations provide an interactive representation of VIBES output, including prophage annotations, bacterial gene annotations and viral gene annotations. An output HTML file is generated for each input bacterial genome, each of which contains interactive annotation visualizations for its associated genome. The HTML files may be opened locally in a web browser, or they may be hosted on a web server. The generated interactive visualizations are described in the ‘Results’ section.

### Pf prophage search

To demonstrate the utility of VIBES as a prophage identification tool, we searched 1072 *Pseudomonas* spp. isolate genomes for integrations of 178 Pf phage variants. *Pseudomonas* spp. genomes were acquired from the *Pseudomonas* Genome Database (v21.1) (49). Some records in the database were renamed to resolve characters that conflict with standard Bash commands, while three records contained no sequence information. Two of the three empty records were populated with data from GenBank, while the third was determined to be redundant and deleted (see Supplementary Data for details on modifications to data acquired from the *Pseudomonas* Genome Database). Phage sequence coordinates for 179 partial or complete Pf prophages were obtained from a study examining Pf prophage lineages (12). One hundred twenty-six *P. aeruginosa* genomes were downloaded using accession IDs provided in the study and the relevant sequences were extracted and assigned identifiers (see Supplementary Data for details). One phage sequence, labeled vs015, contained a substantial insertion that extended the length of the sequence to >70 kb. Such a long query sequence requires a prohibitive amount of memory to search for, so vs015 was removed from our prophage database, leaving a total of 178 Pf phage query sequences derived from 126 *P. aeruginosa* genomes.

Unless otherwise stated, analysis was conducted on the University of Arizona’s Puma HPC cluster on nodes that each contain 94 AMD EPYC 7642 cores and 512 GB of RAM.

### Availability of data and scripts

Supplementary materials documenting scripts and commands used are available at https://github.com/TravisWheelerLab/VIBES-paper.

## Results

### Overview of VIBES output features

#### Detected Pf prophages in bacterial genomes

For each input bacterial genome, VIBES produces a TSV file describing each detected potential prophage sequence in the genome. The TSV fields include matching phage name, match *E*-value, score, match start and end positions on both query (phage) and target (bacterial) sequences, match strand, a match integration ID (see the ‘Prophage search component’ section) and a full-length field populated with True (full length) or False (partial). By default, a match is called full length if it is at least 70% the length of the best-matching prophage sequence, though this parameter can be modified by the user.

#### Bacterial and viral gene annotations

Annotations of genes within bacterial genomes are generated by Prokka with its default annotation databases and settings. For each bacterial genome, full Prokka output is saved and optionally compressed into a zipped tar archive. Annotations of genes within prophage genomes are output in their own TSV format files with fields identical to those produced for prophage annotations except the match ID field, which is excluded for phage gene annotations.

#### Interactive HTML visual output

After each workflow process has completed, VIBES produces the SODA-based HTML visualization files. The interactive representations of the workflow’s output allow users to investigate annotations in a bacterial genome and potential prophages with the following components.

##### Bacterial chromosome plots (Figure 3B)

The visualizations include both circular and linear representations of the bacterial chromosome. Both representations of the chromosome are marked with detected viral integrations (yellow, two outer circles) and bacterial genes (blue, two inner circles) to assist in analysis of integrations and phage landing sites. Hovering over a blue bacterial gene marker displays the name of the gene, while hovering over a yellow phage integration marker displays the name of the prophage. Users can select a viral integration to inspect it more closely (see the ‘Position occurrence plot’ section). Users can zoom in on the chromosome and click and drag to pan across the genome, making gene or integration annotations larger and easier to interact with; simultaneously, the currently visible portion of the genome is highlighted in gray across the chromosome along top of the page. The circular genome can be changed to a linear representation, and vice versa, by clicking the linear button below the interactive chromosome.

**Figure 3.**
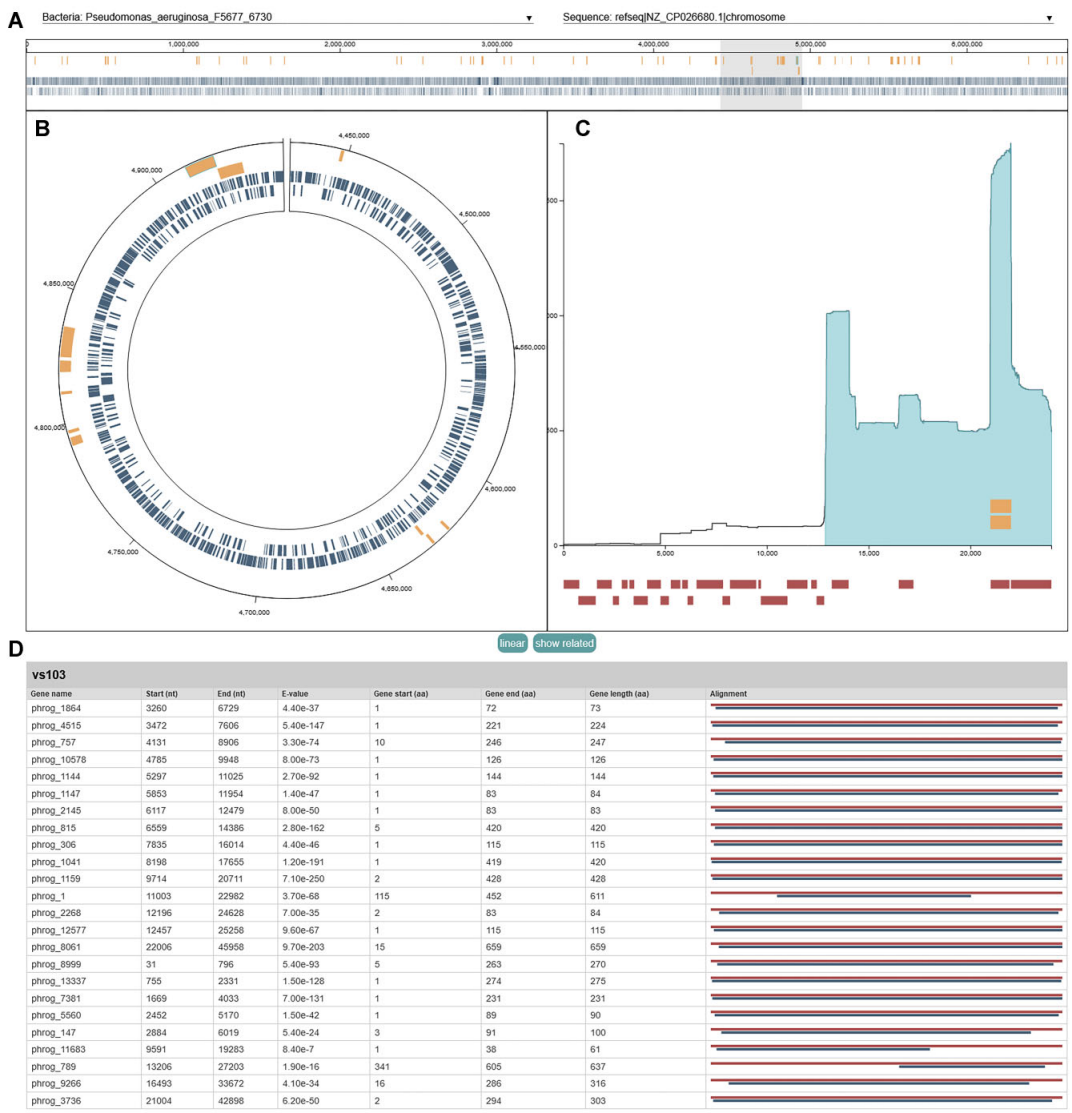
Example VIBES interactive annotation visualization page, displaying a bacterial replicon with gene and prophage annotations, where the selected integration falls on the closest-matching viral genome, and viral gene annotations. (**A**) The full interactive visualization page. (**B**) The bacterial replicon plot includes two modes to represent a selected bacterial replicon: linear and circular, both marked with integration and gene annotations. (**C**) The position occurrence plot displays information about a selected integration, related integrations and prophage gene annotations. (**D**) The query phage gene annotation table contains detailed information about gene annotations on the closest-matching user-provided phage genome.

##### Position occurrence plot (Figure 3C)

To assist users in investigating patterns of phage integration, a position-specific occurrence plot is displayed for a selected integration. The selected integration may be changed by clicking on a corresponding glyph in the genome annotation chart or via the drop-down input at the top of the plot. The *x*-axis of the plot corresponds to each position (nucleotide) in a query phage sequence, while the *y*-axis displays a count at each position summing every occurrence of that position in every integration in the dataset, emphasizing regions of a phage sequence that most often integrate into host genomes. The blue shaded region along the *x*-axis displays the extent of the currently selected integration on the query sequence it matched to. Yellow bars over the *x*-axis show where any other integrations matching to the same query phage on the selected bacterial genome matched to the query, indicating regions of the phage integrated repeatedly into the same genome. The yellow bars indicating where other integrations of the same phage fall on the viral genome can be hidden by clicking the hide-related button located under the position occurrence plot. Under the *x*-axis, red bars display where viral gene annotations fall on the phage genome. Hovering over a viral gene annotation bar shows the name of the gene, while clicking on it highlights its row on the query phage gene annotation table.

##### Query phage gene annotation table (Figure 3D)

At the bottom of the visualization is a table of viral protein-coding gene annotations on query phage sequence most closely matching the currently selected integration. The phage gene annotation table contains a row for each annotated gene displaying the gene name, start and end positions on the query phage genome, annotation *e*-value, start and end positions relative to the reference gene amino acid sequence, and an alignment figure that visually depicts the extent of the match on the query phage sequence (blue line) compared to the reference amino acid sequence (red line).

### Comparison to other prophage annotation tools

To assess performance of the workflow on known prophages, we ran VIBES against two reference *P. aeruginosa* genomes, PAO1 and UCBPP-PA14, which contain Pf4 (50) and Pf5 (51), respectively (Table 2). Both genome sequences were obtained from the *Pseudomonas* Genome Database (49). Pf4 and Pf5 prophages were extracted from their hosts and provided to VIBES as queries. To assess the performance of VIBES relative to other prophage annotation tools, we submitted the genome sequences of PAO1 and UCBPP-PA14 to DBSCAN-SWA and PHASTEST through their web servers. We also ran geNomad (35) via its Docker container on the same HPC system VIBES was run on. All these tools use *de novo* annotation databases to annotate prophage sequences, and so are not directly comparable to VIBES, which depends on the user-provided query library. In this test, VIBES was given the perfect query sequence for each bacterial genome, so the evaluation is primarily useful to understand runtime; in general, VIBES will be as sensitive as the tool it uses to perform sequence alignment (*nhmmer*). We attempted to also submit both genomes to Prophage Hunter, but its web server is no longer available and issues encountered during local installation prevented its assessment.

**Table 2.** Summary tool runtime and results on PAO1 and UCBPP-PA14

Tool	PAO1 runtime (s)	PA14 runtime (s)	PAO1 prophage	UCBPP-PA14 prophage
DBSCAN-SWA	387	414	None	None
geNomad	266	269	1 caudovirus, 1 inovirus	1 inovirus, 1 caudovirus
PHASTEST	536	552	1 intact YMC11, 1 intact Pf1	1 incomplete YMC11, 1 intact Pf1
VIBES	880 (both)	880 (both)	1 full (Pf4)	1 full (Pf5)

VIBES was supplied with Pf4 and Pf5 as prophage queries.

VIBES was run on default settings and set to filter out results <1 kb. VIBES ran its searches in parallel, completing both in 880 s. As expected, VIBES recovers a full-length (13.6kb) Pf4 integration in PAO1 spanning from PA0714 to PA0729.1 (50) and a full-length Pf5 (10.9kb) integration in UCBPP-PA14 spanning from PA14_48870 to PA14_49040 (51). Notably, VIBES did not identify any other sequences in PAO1 or PA14 that matched to Pf4 or Pf5 and were long enough to pass a 1-kb filter. PHASTEST recovered one Pf1 integration, which it ranked intact, in a 15.8-kb window centered on the location of Pf4 in PAO1 and one Pf1 integration, ranked intact, in a 18.2-kb window centered on Pf5 in UCBPP-PA14. In PAO1, geNomad identified one inovirus spanning from PA0716.1 (*pf4r*) to PA0728.1 (*pfiA*) with a length of 9 kb, while in UCBPP-PA14 geNomad identified one inovirus with a length of 11 kb spanning from PA14_48850 to PA14_49010. DBSCAN-SWA did not detect prophages in either genome, but this could occur if its *de novo* annotation database does not contain queries homologous to Pf4 or Pf5.

### Application to *Pseudomonas* dataset

To explore the utility of the VIBES workflow for identifying (possibly fragmented) phage integrations within bacterial isolates, we applied the workflow to a dataset composed of 1072 publicly available *Pseudomonas* spp. genomes obtained from the *Pseudomonas* Genome Database (v21.1) (49) and 178 Pf phage variants published in a study on Pf phage lineages (12). Nextflow reported that the prophage detection component of the workflow consumed 13 526.3 CPU hours across 2099 tasks in its prophage search component, 398.8 CPU hours across 1072 tasks in its bacterial gene annotation component and 49.7 CPU hours across 539 tasks in its viral gene annotation component, totaling 13 974.8 CPU hours consumed across a total of 3710 tasks (more details on resource usage can be found in Table 3).

**Table 3.** Resource usage

Component	Total CPU hours	Tasks run	Most expensive process	CPUs allocated	Mean RAM	Mean runtime (min)
Prophage search	13 526.3	2099	*nhmmer*	2	36.8 GB	412.5
Bacterial gene annotation	398.8	1072	Prokka	6	901.2 MB	3.5
Viral protein annotation	49.7	539	BATH	2	403.5 MB	8.6

CPUs allocated, mean RAM and mean runtime all display values for the most expensive process in each component, as the computational cost of other elements was negligible. GB stands for gigabyte and MB stands for megabyte.

**Figure 4.**
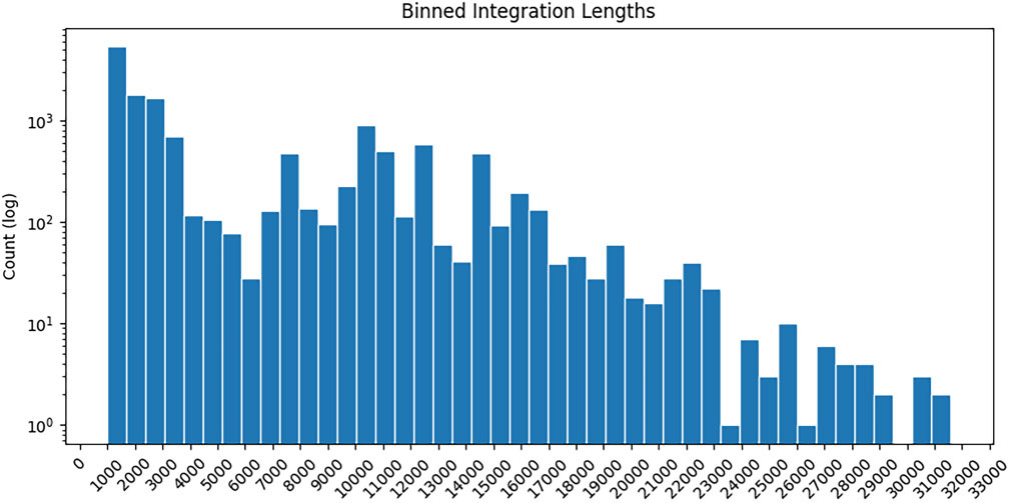
Integration length bin plot. Counts of likely integrations binned by length, where joined integrations were summed together. The *y*-axis uses a log scale due to the large number of short integrations identified.

**Figure 5.**
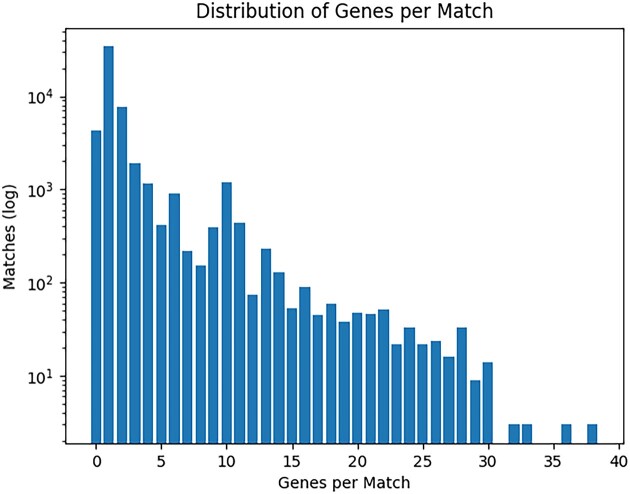
Distribution of genes per match. Each bar represents how many matches (among the 51 903 total initially reported by the pipeline) mapped to a region on the query prophage annotated with the number of genes labeled on the *x*-axis. Thirteen thousand eight hundred fifty matches mapped to regions annotated with at least two viral protein-coding genes.

In our input dataset of 1072 target *Pseudomonas* spp. genomes and 178 query Pf phage variants, VIBES reported 517 full-length Pf phage integrations and 51 386 partial hits. Of the 51 903 hits identified, 1398 were made up of two or more joined fragments. The vast majority of reported hits were <1500 nucleotides in length. To reduce the incidence of short, ambiguous hits (which may have been matches to homologous bacterial genes or to regions of bacterial genome erroneously included on the terminal ends of prophage queries), we discarded any hit that mapped to a query prophage region containing <2 genes annotated by the pipeline’s viral protein-coding gene component. The per-match gene count filter reduced the number of partial hits from 51 386 to 13 850 likely integrations with a mean and median length of 5095 and 2404 nucleotides, respectively (Figure 4). A histogram of gene counts per likely integration is available in Figure 5.

## Discussion

Here, we have introduced a new software package that conveniently manages the workflow of annotating an arbitrarily large number of bacterial genomes, with special emphasis on identification of prophages. The VIBES workflow produces output annotation TSVs that are easy to load into a spreadsheet viewer or parse programmatically, along with helpful interactive HTML visualizations that facilitate analysis of identified prophages, their landing sites and protein-coding genes on query phage sequences. VIBES also provides a basic framework for the management of general-purpose, large-scale annotation projects.

### Recommended usage

VIBES is best suited to conduct large-scale analysis of relatively well-characterized prophages in cluster or cloud computing environments where parallelization of search processes yields the largest gains, and where the impact of its convenience features (prerequisite management with Docker/Singularity and job orchestration and automated job management via Nextflow) is most pronounced. We recommend that users source query prophage sequences from manually curated databases when possible, to avoid odd phage–bacteria boundary effects that can occur during *de novo* annotation. If users instead use predicted prophage regions as queries, we recommend that they use a higher minimum hit length threshold and make use of a script available in the VIBES repository, which filters out matches that do not contain an adjustable number of viral genes. VIBES is also well suited to large-scale analyses of homologous sequences shared between prophages and host genomes, given the high sensitivity of *nhmmer* search to distant homology (40) relative to other sequence similarity search software.

VIBES may also be useful as a secondary step in *de novo* prophage annotation. Other tools, such as PHASTEST (36), are well suited to identifying unknown prophage integrations, but provide relatively coarse annotations. Once integrations have been coarsely identified and users have a notion of what prophage sequences to supply as queries, VIBES is relatively well suited to more precisely determine prophage strain and integration boundaries if prophage regions are provided as target sequences.

### Alternative use of the VIBES workflow

To a large degree, VIBES is a workflow that coordinates sequence similarity search and bacterial genome annotation when supplied with target and query sequences. The only VIBES components that are specific to annotation of prophage in bacterial genomes are Prokka (37) annotation (which requires bacterial sequence input) and visualization generation (which assumes that targets are bacterial genomes and queries are prophages). The core input to VIBES is simply target sequences (in FASTA format) and queries (in either multi-FASTA for a set of sequences or Stockholm for a set of alignments). As a result, users are essentially free to use VIBES to conduct searches against nucleotide sequences as they see fit, so long as they specify query sequence type (DNA, RNA or amino) and disable incompatible workflow components through the VIBES configuration file.

### Future extensions

Here, we briefly discuss some limitations of the VIBES workflow that could be addressed by future extensions of the workflow. These limitations are discussed in more detail in the VIBES documentation, available at https://github.com/TravisWheelerLab/VIBES.

#### *Att* site annotation

A common signal of phage integration is the presence of attachment (*att*) sites, two copies of which—*attP* and *attB*, carried on the phage genome and bacterial genome, respectively—flank integrated prophages. These leave a distinctive signal of flanking direct repeats, but this signal is not detected or annotated in the initial VIBES release. As *att* sites are of interest to prophage researchers and are annotated by some prophage annotation tools (26,52), extending the VIBES feature set to include *att* site annotation is a high priority for future extensions of the workflow.

#### Further prophage verification

A major limitation of the VIBES workflow is its limited means of verifying that query prophage sequence represents a complete or partial prophage sequence, rather than a homologous non-phage region of the host genome. Currently, VIBES employs relatively simple methods of analyzing ambiguous sequences via its interactive visual output, its complete/partial classification and its per-match gene count filtering. While this may be desirable in some analyses, such as investigations of homologous regions shared by prophages and their hosts, addressing this limitation is a high priority for future iterations of the workflow.

#### *De novo* annotation support

The default VIBES workflow does little to support *de novo* annotation of phage sequences, in which bacterial genomes are scanned for a broad range of reference prophages. Currently, to conduct such an analysis with VIBES, users would have to identify or construct a file containing a diverse set of known temperate phage genomes or multiple sequence alignments of related phage genomes. *De novo* annotation is further hampered by the lack of further analysis of potential prophages identified by VIBES, discussed above, which makes it more difficult to discern prophage regions from homologous bacterial sequences, especially when given a diverse set of temperate phage queries.

#### Support for other search tools

Though the *nhmmer* search algorithm is more sensitive to distant homology than other search algorithms (40), it is relatively computationally expensive. Indeed, viral sequence annotation tools that use HMMER search algorithms have been demonstrated to be among the slowest annotation techniques (53). This limitation can be addressed through the addition of a broader choice of sequence similarity search tools with less expected sensitivity and resource usage, such as LAST (54) or *blastn* (44).

## Data Availability

Supplementary materials documenting scripts and commands used are available at https://github.com/TravisWheelerLab/VIBES-paper, https://zenodo.org/doi/10.5281/zenodo.10826544 (VIBES software) and https://zenodo.org/doi/10.5281/zenodo.10826552 (scripts/notes for article figures/tables).
